# Synergistic Effects of Simulated Energy Drink Exposure and Fatigue Loading on Bioactive and Conventional Resin Composites

**DOI:** 10.3390/jfb17010029

**Published:** 2026-01-03

**Authors:** Fatin A. Hasanain, Alaa Turkistani

**Affiliations:** Department of Restorative Dentistry, Faculty of Dentistry, King Abdulaziz University, Jeddah 21589, Saudi Arabia; aturkistani1@kau.edu.sa

**Keywords:** dental resin composite, energy drink simulation, cyclic fatigue, flexural strength

## Abstract

The consumption of energy and sports drinks is on the rise globally, exposing dental restorations to more frequent low-pH challenges, which affect degradation. This in vitro study simulated the combined effect of energy drink exposure and cyclic fatigue loading on the fatigue survival rate and flexural strength of three direct dental resin restorative materials with distinct chemistries: a bioactive ionic resin (Activa Presto), a giomer (Beautifil Flow Plus F00) and a conventional nano-hybrid composite (Tetric Ceram). Bar-shaped specimens (25 × 2 × 2 mm) were fabricated according to ISO 4049 and stored for 24 h in either distilled water or 0.2 M citric acid (pH ≈ 2.5), simulating an energy drink (*n* = 10/group). The samples then underwent chewing simulation (40 N, 100,000 cycles, 1.6 Hz) using a steel antagonist; surviving specimens were tested via three-point bending to determine their flexural strength. All the materials were affected by storage conditions: Activa Presto showed the lowest fatigue survival (20% in water; 0% in citric acid), Tetric N-Ceram moderate survival (40% in both solutions) and Beautifil Flow Plus F00 the highest and most stable survival (90% in water; 40% in citric acid). Among the surviving specimens, Tetric Ceram exhibited the highest flexural strength, followed by Beautifil Flow Plus F00 and then Activa Presto. Citric acid exposure and cyclic loading adversely affected the mechanical performance of all the materials within the limitations of this study.

## 1. Introduction

In most dental practices, dental resin composites are a mainstay of direct restorations due to their pleasing esthetics and constantly improving mechanical properties [1,2,3,4,5]. However, dental resin composites and other direct filling materials remain vulnerable to both chemical and mechanical degradation once placed into the oral cavity. In the complex and dynamic oral environment, restorative materials are exposed to repeated thermal changes, moisture, enzymatic activity and mechanical loading. The mechanical fatigue caused by both mastication and parafunctional habits can initiate microcrack formation and reduce longevity [6,7]. Chemical challenges, especially from acidic beverages, have been found to have negative effects on the resin matrix and filler–matrix bond [8,9].

Currently, dental patients expect esthetic, minimally invasive restorations that are durable regardless of changing dietary habits. One such trending habit is the increasing energy and sports drink consumption, especially among young adults and adolescents. These drinks are marketed as performance optimizers and have perceived lifestyle benefits, with global estimates suggesting that more than half of young people regularly consume them [8,10]. Prolonged sipping of such beverages extends the duration of acidic exposure. As energy and sports drinks become a routine part of life for a larger population, their effect on restorative materials has become more of a concern for practitioners. The reason for concern is that these beverages have low pHs, with average values between 2.5 and 3.5, typically due to their citric acid content, which can erode enamel and degrade resin-based materials [11]. Citric acid can weaken composite structure and accelerate its degradation, acting as a chelating agent and a proton donor capable of both disrupting silane coupling and dissolving filler particles in dental resin composites [12]. As recall intervals lengthen and heavy functional demands are placed on resin composites, their long-term behavior in erosive environments becomes a critical aspect of clinical decision-making. Citric acid was selected as a standardized acidic challenge to represent a key erosive component of energy drinks.

The mechanism of degradation for dental resin composites is a combination of enzymatic, mechanical and hydrolytic mechanisms over time. The filler–matrix interface is disrupted due to an increase in polymer chain mobility caused by the materials’ water sorption [9,10], while exposure to acid catalyzes ester hydrolysis and weakens the silane coupling layer, which is essential for stress transfer between the resin matrix and inorganic fillers [11]. Thus, even a moderate pH drop can have an influence on mechanical properties such as flexural strength and fatigue resistance [6]. As energy drinks often have pH levels ≤ 3, following their consumption, the oral environment may present a substantial challenge to restorative materials, especially with habitual or repeated drinking. It is also worth noting that dental restorative materials do not respond uniformly to oral challenges, as their formulations differ in terms of resin chemistry, filler composition and polymer network hydrophilicity. All of these factors affect the mechanism of degradation [1,10].

The use of bioactive and giomer restorative materials has increased in parallel with this increase in energy drink consumption. The term bioactive is used when dental restorative materials do more than simply restore teeth to function. These materials produce an additional beneficial effect [4]. These dental materials were introduced to remineralize the remaining tooth structure rather than remain inactive after placement, with bioactive dental resins releasing fluoride, sometimes in combination with other materials aimed at tooth remineralization [13,14]. Giomers are a hybrid of dental resin composite and glass ionomers. They utilize surface pre-reacted glass (S-PRG) particles in their matrix to combine the strength of conventional dental composites with fluoride release [15]. Both these materials incorporate hydrophilic resin matrices and ion-exchange mechanisms, which may reduce their resistance to chemical degradation, with studies finding that both giomers and bioactive resins show reductions in mechanical properties after exposure to acidic environments [16]. Nano-hybrid dental resin composites contain hydrophobic resin matrices, dense cross-linking and higher filler loads, which enhance their resistance to acid attacks [17]. While previous studies have explored the chemical or mechanical degradation of these materials by exposing them to an acidic challenge or mechanical fatigue, few have assessed both conditions concurrently. However, these conditions occur simultaneously in the oral cavity. Thus, this study investigated the combined effect of simulated energy drink acidity and cyclic fatigue loading on the fatigue survival and flexural strength of three dental resin filling materials: Activa Presto, Beautifil Flow Plus F00 and Tetric Ceram.

The null hypotheses were that (1) neither citric acid conditioning nor material type would affect fatigue survival; (2) flexural strength would not differ among materials or conditioning environments.

## 2. Materials and Methods

The materials used in this work are shown in Table 1.

### 2.1. Specimen Fabrication

In accordance with ISO 4049:2019 [18], all the samples were formed into 25 × 25 × 2 mm bars using a stainless-steel split mold (New Age Research USA, Miami, FL, USA). The mold was placed on top of a Mylar strip on a flat surface, the material was inserted, and a second Mylar strip and a glass slide were placed over the filled mold. Finger pressure was used to extrude any excess material. The sample was then cured in overlapping circles using an Elipar LED light-curing unit (3M ESPE Elipar, St. Paul, MN, USA) to ensure a complete cure and avoid undercuring, which was an important factor in the study. The light-curing unit was in direct contact with the Mylar strip (0 mm distance), and curing time was as per manufacturers’ instructions. The power density of the light-curing unit was measured with a handheld radiometer (Bluephase Meter II, Ivoclar, Amherst, NY, USA) at 1200 mW/cm^2^ prior to specimen preparation.

Each material’s set of samples was then divided into two groups:

1. Citric acid group: 10 samples per material (*n* = 10) stored in 0.2 M citric acid (pH ≈ 2.5) at 37 °C for 24 h to simulate energy drink acidity [8,9].
2. Distilled water: 10 samples per material (*n* = 10) stored in distilled water at 37 °C for 24 h.

### 2.2. Fatigue Loading

**Figure 1:**
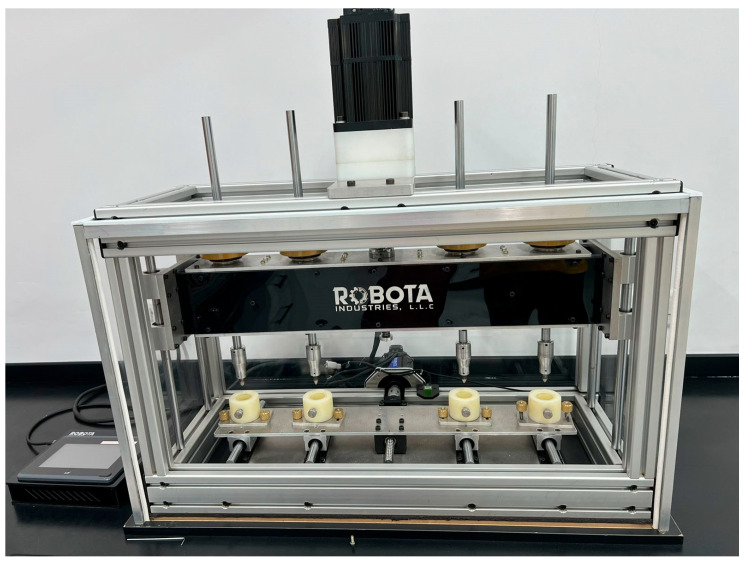
Robota chewing simulator.

Once samples were removed from storage, they were placed in a custom-made chewing simulator (Robota chewing simulator, New Borg AlArab, Alexandria, Egypt), as shown in Figure 1, and subjected to 100,000 chewing cycles at 40N load with a steel cusp antagonist. Any samples that survived fatigue loading were then subjected to flexural strength testing.

### 2.3. Flexural Strength Testing

**Figure 2:**
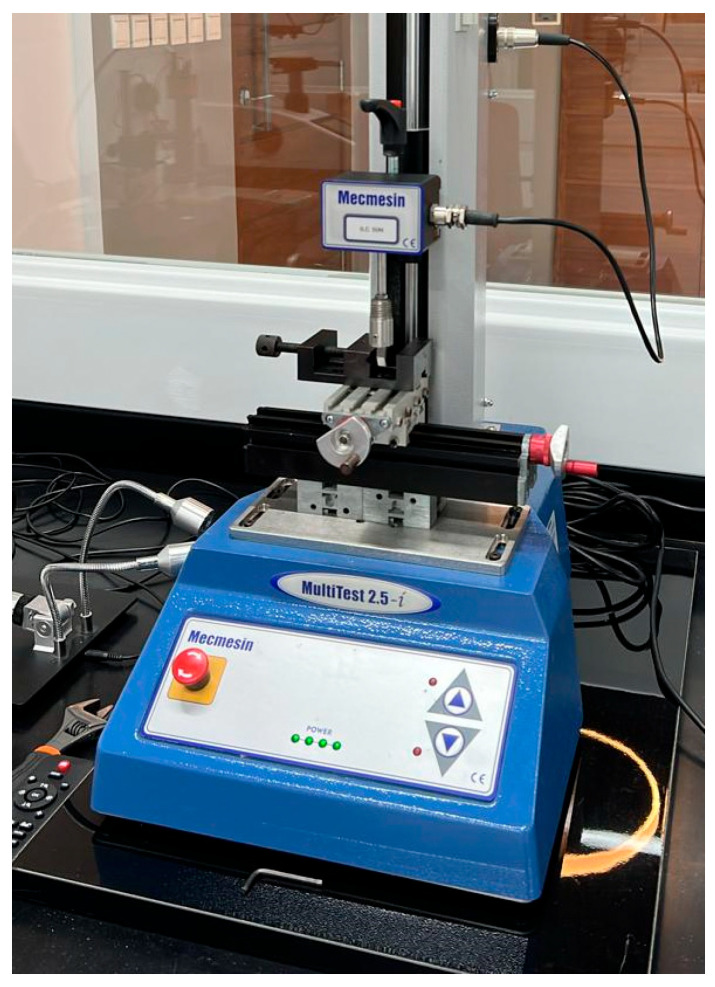
Universal Testing Machine.

Surviving specimens were tested using the Universal Testing Machine (Mecmesin MultiTets 2.5-i, Mecmesin (PPT Group UK Ltd), Slinfold, West Sussex, UK) with a crosshead speed of 1 mm/min (Figure 2). Three-point bending tests were performed in accordance with ISO 4049:2019.

### 2.4. Statistical Analysis

Calculations made with G*Power software (version 3.1.9.7, Germany) indicated that a sample size of *n* = 10/group would yield approximately 80% power to detect large differences in failure rates. Thus, 10 samples were fabricated per group.

Statistical analyses were performed using IBM SPSS Statistics (Version 29.0, IBM Corp., Armonk, NY, USA). Fatigue survival was analyzed as a binary outcome using Fisher’s exact test. Survival differences among the three materials within each solution were examined using chi-square tests, followed by Fisher’s exact test for pairwise comparisons when needed.

Only specimens that survived the fatigue testing were included in the flexural strength analysis, performed using one-way ANOVA for materials stored in distilled water. It was then followed by Tukey’s post hoc test for multiple comparisons. For specimens conditioned in citric acid, a one-way ANOVA was conducted between Beautifil and Tetric N-Ceram only, as none of the Activa specimens survived in citric acid. Cycles-to-failure data were summarized descriptively (mean ± SD) for fractured specimens; no other statistics were analyzed for this outcome due to unequal group sizes and the presence of single-specimen groups. Statistical significance was set at *p* < 0.05.

## 3. Results

### 3.1. Fatigue

Fatigue survival differed by both material and storage solution, as shown in Table 2. When comparing the same material in different solutions, none of the materials showed a significant difference. None of the Activa Presto samples immersed in citric acid survived fatigue testing.

When comparing different materials in the same solution, the groups kept in water showed a significant difference. Beautifil F00 had a significantly higher survival rate when compared to Activa Presto (*p* = 0.0055), while there was no significant difference between Activa Presto and Tetric N-Ceram (*p* = 0.6285). When comparing the acid-stored groups, no statistically significant differences were found. Overall, Beautifil F00 exhibited the highest fatigue survival, particularly after water storage, whereas Activa Presto consistently showed the lowest survival rate. All these results are further detailed in Table 3 and Table 4.

### 3.2. Flexural Strength 

Only the specimens that survived fatigue loading had their flexural strength measured, and descriptive statistics are shown in Table 5. No flexural strength measurements were obtained for Activa Presto, as none of those samples survived fatigue testing.

Comparison of the same material in different solutions showed that Beautifil F00 survivors stored in water had significantly greater flexural strength than those stored in acid, whereas there was no significant difference found between water and acid storage survivors for Tetric N-Ceram. No flexural strength comparison between solutions was possible for Activa Presto, as no acid-stored specimens survived fatigue testing.

One-way ANOVA showed that there were significant differences in the surviving water-stored samples between materials. Post hoc testing revealed that the Beautifil F00 specimens had a significantly higher flexural strength than Activa Presto, while Tetric N-Ceram had a significantly higher flexural strength than Beautifil F00. Among the acid-stored samples, only Beautifil F00 and Tetric N-Ceram were tested, as there were no surviving Activa Presto specimens. Tetric N-Ceram showed significantly higher flexural strength than Beautifil F00. These results are detailed in Table 6.

## 4. Discussion

The present study investigated the effect of both simulated energy drink acidity and cyclic fatigue on the performance of three resin-based restorative materials with distinct chemical formulations: a bioactive ionic resin material (Activa Presto), a giomer (Beautifil Flow Plus F00) and a conventional nano-hybrid dental resin composite (Tetric N-Ceram). These materials were chosen so as to represent what is available to dental practitioners, and in our tests, the use of 40 N and 100,000 cycles simulated the load on a posterior restoration for approximately six months [19]. The chewing simulator used a stainless-steel cusp antagonist. That antagonist provides stable and reproducible contact conditions during fatigue loading since manufactured, hard antagonists have been shown to maintain more consistent abrasive behavior and surface characteristics throughout laboratory wear and fatigue simulations [20]. The use of natural enamel antagonists is inherently variable in the antagonists themselves due to enamel’s biological variability, which can in turn result in inconsistent wear behavior for both antagonist and sample during laboratory testing. Enamel is a brittle material, and its surface conditions may change under cyclic loading, further compromising the reproducibility of the test [21]. Consequently, stainless-steel antagonists are commonly employed in fatigue and wear simulations when the aim is to evaluate the behavior of restorative materials under controlled loading conditions while minimizing variability introduced by the antagonist itself. The first null hypothesis was rejected, as the results showed that both material type and acid conditioning had an effect on fatigue survival, with material type showing a significant difference. Flexural strength was also significantly affected by immersion solution and material type, and thus the second hypothesis was rejected.

The fatigue survival of all of the tested materials was a noteworthy finding. Beautifil Flow Plus F00 showed the highest fatigue survival in water while maintaining a moderate survival rate after immersion in citric acid. On the other hand, Activa Presto showed the lowest survival rate in the acid-immersion group, with no samples surviving fatigue testing, thus giving it the lowest survival rate of the materials tested. This complete loss of specimens after acid immersion may be due to the acid weakening the filler–matrix bond and ion-leachable glass fillers prior to fatigue testing. Tetric N-Ceram had similar fatigue survival in both storage environments, yet showed the highest flexural strength among the surviving specimens.

Even though no significant differences were observed when comparing the immersion solutions of the materials tested, directional trends could be observed. Regardless of material type, materials immersed in citric acid showed a lower fatigue survival rate, suggesting that the acid conditioning may have had an effect that the sample size was not large enough to show. From a clinical perspective, such trends are relevant findings, as even minor performance reductions could lead to earlier restoration failure. Acid exposure has been shown to have an adverse effect on restorative materials due to acid’s chelating properties on the resin matrices and filler–matrix interface [11,22,23]. The results found in this work align with those seen in previous studies regarding the decrease in restoration fatigue resistance when exposed to acidic environments [17].

The higher flexural strength and relative stability of Tetric N-Ceram may be attributed to its hydrophobic resin system composed of Bis-GMA/Bis-EMA/UDMA in combination with densely packed silanated fillers. Hydrophobic matrices limit water diffusion, which in turn decreases degradation in these materials by reducing plasticization and ester bond hydrolysis [9,10]. On the other hand, hydrophilic matrices exhibit greater water sorption and hydrolytic vulnerability [10].

The most pronounced degradation was seen in Activa Presto, especially after immersion in acid. Activa Presto is a bioactive ionic resin material made to release calcium, phosphate and fluoride ions from an ion-leachable glass system through a relatively hydrophilic resin matrix [24]. While this formulation aims to aid in tooth structure remineralization, it increases the material’s susceptibility to water sorption, filler–matrix debonding and hydrolytic degradation, especially after immersion in acidic media [10,11]. The results shown in this work are consistent with previous research, which has shown a substantial reduction in bioactive dental composites after exposure to acidic media [17], even though others have shown an improvement after acid immersion [16]. The difference in results may be dependent on exposure duration and pH cycling protocol, as well as the absence of mechanical stress after acid immersion.

As mentioned earlier, the giomer restorative material used, Beautifil Flow Plus F00, incorporates surface pre-reacted glass (S-PRG) fillers engineered to provide fluoride and multifunctional ion release [15]. When exposed to low pHs, the S-PRG fillers also undergo ion exchange and dissolution, altering their surface morphology and causing weakening at the filler–matrix interface [25]. This mechanism may explain the reduction in flexural strength observed after citric acid conditioning, consistent with previous findings that giomers soften and show decreased hardness after exposure to erosive beverages [25,26]. The clinical implications of these degradation mechanisms are discussed later in this section.

The material-dependent influence of acid immersion can be seen clearly in the differences in flexural strength between water- and citric-acid-conditioned specimens. In Beautifil Flow Plus F00, the significant flexural strength reduction in the acid-immersed specimens suggests that the giomer matrix and filler system are both susceptible to acid-mediated degradation. No significant difference was found in Tetric N-Ceram when comparing storage media, possibly due to the hydrophobic nature of its matrix and densely silanated fillers. Even though Activa Presto could not be evaluated after acid storage due to complete fatigue failure, the reduced flexural strength in water-stored survivors further supports the vulnerability of ion-releasing materials to hydrolytic and chemical degradation. Overall, these findings indicate that exposure to an acidic environment compromises flexural strength, especially in materials with reactive or hydrophilic components. Nano-hybrid dental resin composites were found to demonstrate comparatively greater mechanical stability, even though there was a slight dip in the flexural strength measured. This is in agreement with previous work on nano-filled dental resin composites [27]. In order to improve resistance to acidic environments, material design strategies such as increased resin matrix hydrophobicity, improved filler–matrix interfacial stability, and reduced hydrolytic susceptibility could all be employed in future formulations.

Using a combination of both mechanical and simulated environmental challenges, this work attempted to approximate what happens in the oral cavity more comprehensively than testing either factor alone. This is a clinically relevant combination, as acidic beverages are frequently consumed between or during meals, thus creating a cycle in which the pH is lowered and mastication then occurs.

This work also highlights the importance of patients’ diet analysis in order to choose the most durable restoration, as dietary habits play a critical role in the long-term performance of resin-based restorations and treatment planning in general [28,29]. Incorporating dietary assessment into routine restorative decision-making may help optimize material selection, improve restoration longevity and reduce the need for premature replacement in high-risk patients [30]. A detailed assessment of a patient’s dietary habits, including but not limited to the frequency and duration of exposure to acidic food, provides information that is neither available upon examination of the oral cavity nor inferable from oral findings alone [31,32]. High-frequency acidic intake is a recognized risk factor for erosive tooth wear and restoration longevity for resin-based and glass-containing restorative materials [33,34,35], and identification of such behavioral patterns would also help clinicians target interventions, should they be required [36]. The incorporation of dietary analysis into routine clinical decision-making aims to create a risk-based approach to dental care that addresses etiology as well as clinical consequences, thereby improving restoration longevity and long-term health outcomes [30].

The clinical implications of these findings are relevant to dental practitioners. With the rise in energy and sports drink consumption in adolescents and young adults, the risk of both erosion and new carious lesions increases [8,37,38,39]. These beverages have low pHs along with sugars and carbonation that further prolong their erosive potential [8,22]. The findings from this study, though preliminary, will hopefully guide dental clinicians to assess their patients’ diet prior to placing permanent restorations and weigh the factors at play before making a final informed decision. Based on the findings from this work, patients with frequent exposure to acidic beverages may have more predictable longevity with nano-hybrid composites as the restorative material of choice, and giomers and bioactive ionic materials may require cautious placement or more frequent monitoring in such cases.

As with any scientific work, this study has several limitations. Firstly, it is a laboratory in vitro study, and no matter how many factors are included, no in vitro study will fully mimic the oral cavity. However, factor isolation is one way to understand material behavior. Another limitation is the use of ISO bar-shaped specimens. While they were used to facilitate specimen standardization and ensure that flexural strength testing could be performed on the surviving samples, the bar-shaped specimens did not replicate the geometry or occlusal loading pattern found in a clinical cavity. Additionally, their high surface-to-volume ratio may have exaggerated specimen degradation during testing [1,9]. It is also worth noting that in the oral cavity, saliva buffers any change in the oral environment, whereas continuous acidic immersion simulates an intentionally aggressive protocol without salivary buffering [40]. Thus, the testing protocol followed in this work is a worst-case scenario, and the findings are interpreted relatively. There are several other factors in the oral cavity that affect restorative materials that were not included in this work, such as the different temperatures of consumed food and drink, which can be investigated via thermocycling; pH cycling was also not included. The low fatigue survivor count in certain groups also limited statistical comparisons. It must be noted, however, that this study’s methodology aligns in principle with that used in previous work on dental resin restorative material fatigue and wear [27,41,42].

Future research can help overcome some of the limitations mentioned. Conducting these experiments on more samples would enable researchers to see even smaller effects of acid immersion. Future work can also incorporate more factors, including buffering in between pH-immersion cycles, thermocycling and larger numbers of cycles. Identifying which material characteristics most strongly predict resistance to combined chemical and mechanical degradation will be essential for guiding next-generation material design.

## 5. Conclusions

Within the limitations of this study, the following conclusions could be drawn:

1. Citric acid exposure, simulating energy drink acidity, reduced the fatigue survival and flexural strength of resin composites.
2. These effects were material-dependent: Activa Presto and Beautifil Flow Plus F00 were more affected than Tetric N-Ceram.
3. Tetric N-Ceram exhibited superior stability under acidic conditions.
4. Frequent consumption of acidic beverages may compromise the longevity of bioactive and giomer restorations.

## Figures and Tables

**Table 1:** Materials used and their formulations.

Material Name Lot Number	Manufacturer	Resin(s) Used	Filler(s)
Beautifil Flow Plus F00 Lot: 082271	Shofu Inc. (Kyoto, Japan)	Urethane dimethacrylate (UDMA), other monomers (not specified)	Surface pre-reacted glass ionomer (S-PRG) 75% by weight
Activa Presto Lot: 221129	Pulpdent corp. (Watertown, MA, USA)	UDMA, other monomers (35%)	Ion-leachable glass 70% by weight
Tetric N-Ceram Lot: Z06P6D	Ivoclar (New York, NY, USA)	Bisphenol A-diglycidyl dimethacrylate (Bis-GMA) Ethoxylated bisphenol A dimethacrylate (Bis-EMA) UDMA	Barium glass Ytterbium trifluoride mixed oxide Pre-polymerized filler (prepolymers) (56% vol.)

**Table 2:** Results of Fisher’s exact tests comparing survival outcomes between storage solutions for each material.

Material	Solution	Surviving Samples	*p*-Value
Activa Presto	Water	2	
Activa Presto	Citric Acid	0	0.474
Beautifil F00	Water	9	
Beautifil F00	Citric Acid	4	0.057
Tetric N-Ceram	Water	4	
Tetric N-Ceram	Citric Acid	4	1.000

**Table 3:** Survival comparison among materials within each storage condition (chi-square tests).

Storage Condition	Material	X^2^	*df*	*p*-Value
**Water**	Activa Presto	10.40	2	0.0055
	Beautifil F00			
	Tetric N- Ceram			
**Citric Acid**	Activa Presto	5.45	2	0.065
	Beautifil F00			
	Tetric N-Ceram			

**Table 4:** Post hoc testing (Fisher’s exact test).

Material Pairwise Comparison	Water	Citric Acid
Activa Presto vs. Beautifil F00	0.0055	0.0870
Activa Preston vs. Tetric N-Ceram	0.6285	0.0867
Beautifil F00 vs. Tetric N-Ceram	0.0573	1.000

**Table 5:** Descriptive statistics for flexural strength testing.

Material	Solution	n	Mean (N)	SD (N)	Median (N)	Min (N)	Max (N)
Activa Presto	Water	2	10.52	1.11	10.52	9.73	11.30
Activa Presto	Acid	0	–	–	–	–	–
Beautifil F00	Water	9	20.05	5.19	20.00	13.70	27.25
Beautifil F00	Acid	4	11.25	0.90	11.50	10.00	12.00
Tetric N-Ceram	Water	4	28.07	3.90	28.80	22.68	32.00
Tetric N-Ceram	Acid	4	22.66	1.42	22.99	20.66	24.00

**Table 6:** Flexural strength comparison.

Comparison Type	Group(s) Compared	*p*-Value
Between solutions (water vs. acid)	Activa Presto	—
	Beautifil F00: Water vs. Acid	0.0028
	Tetric N-Ceram: Water vs. Acid	0.20
Between materials (water only)	Activa Presto vs. Beautifil F00	0.036
	Activa Presto vs. Tetric N-Ceram	0.133
	Beautifil F00 vs. Tetric N-Ceram	0.020
Between materials (Citric acid only)	Beautifil F00 vs. Tetric N-Ceram	0.0286
	Activa Presto vs. Others	—

## Data Availability

The original contributions presented in the study are included in the article, further inquiries can be directed to the corresponding author.
